# Seismic imagery from volcanoes on the Azores Plateau implies that explosive deep-water eruptions are more common than previously thought

**DOI:** 10.1038/s41598-026-53050-0

**Published:** 2026-05-13

**Authors:** Christian Hübscher, Annalena Friedrich, Jonas Preine, Christoph Beier, Anthony Hildenbrand, Paraskevi Nomikou, Pedro Terrinha, Benedikt Weiß

**Affiliations:** 1 Department of Earth System Sciences, University of Hamburg, Hamburg, Germany; 2 National Oceanography Centre (NOC), Southampton, UK; 3 Department of Geology and Geophysics, Woods Hole Oceanographic Institution, Woods Hole, MA USA; 4 Department of Geosciences and Geography, Research Programme of Geology and Geophysics (GeoHel), University of Helsinki, Helsinki, Finland; 5 GEOPS, Université Paris-Saclay, CNRS, Orsay, France; 6 Department of Geology and Geoenvironment, National and Kapodistrian University of Athens, Athens, Greece; 7 Department of Marine Geology and Georesources, Portuguese Institute for the Sea and Atmosphere (IPMA), Lisbon, Portugal; 8 Instituto Dom Luiz (IDL), Lisbon, Portugal; 9 Bundesamt Für Seeschifffahrt Und Hydrographie (BSH), Hamburg, Germany

**Keywords:** Submarine volcanism, Deep-water explosive eruptions, Shift in eruption style, Azores Plateau, Lava flows, Seismic stratigraphy, Natural hazards, Solid Earth sciences

## Abstract

It is generally assumed that volatile-rich magmas erupt predominantly effusively at water depths of several hundred meters, as the high hydrostatic pressure suppresses explosive activity. This view is supported by numerous multibeam surveys showing that most submarine volcanoes lack explosive craters. Here, we present high-resolution reflection seismic and bathymetric data from submarine volcanoes located at more than 2 km water depth along the southern transtensional diffuse plate boundary of the Azores Plateau. The seismic reflection characteristics of the investigated edifice flanks resemble those of volcanoes elsewhere that have been verified by drilling to consist of highly fragmented volcaniclastic deposits, including ash, lapilli or pumice. Additional evidence for explosive activity comes from a crater that was subsequently filled with mounded coarser volcanic deposits, or even effusive lava, during the waning stages of eruption. Consequently, earlier multibeam studies were unable to detect indicators for explosive eruptions. Our results demonstrate that submarine volcanic morphology alone is insufficient to rule out explosive eruptions and highlight the value of seismic imagery to systematically identify explosive submarine volcanism. Deep volcanic explosions seem more common than generally admitted, even on oceanic plateaus. This has implications for magmatic volatile outputs and their regional to global environmental impacts.

**Supplementary Information:**

The online version contains supplementary material available at 10.1038/s41598-026-53050-0.

## Introduction

More than one million submarine volcanoes are thought to exist globally^1^; yet, the processes that initiate and shape deep-water volcanoes (DWVs; water depth > 300 m below sea level (mbsl)^2^ or > 500 mbsl^3^) remain poorly understood. Access to these remote systems is limited, especially in the deep ocean, and much of their eruptive behavior is inferred indirectly by multibeam, dredging or short sediment cores. It is generally assumed that at water depths of several hundred meters, even volatile-rich silicic magma erupts mainly effusively because high hydrostatic pressure suppresses volatile expansion^2–4^. This interpretation appears consistent with bathymetric surveys, which show that most submarine edifices lack explosion craters^5–9^. However, the deposits of explosive eruptions have been documented at depths of several kilometers^10–13^, in agreement with experimental and numerical models indicating that explosive behavior can persist at pressures equivalent to > 3 km water depth^14–17^.

Whether submarine volcanoes can shift from explosive to effusive activity during a single eruption—through progressive degassing, as known from subaerial systems^18,19^—remains largely unexplored because submarine deposits are rarely accessible for direct sampling or stratigraphic reconstruction.

**Fig. 1:**
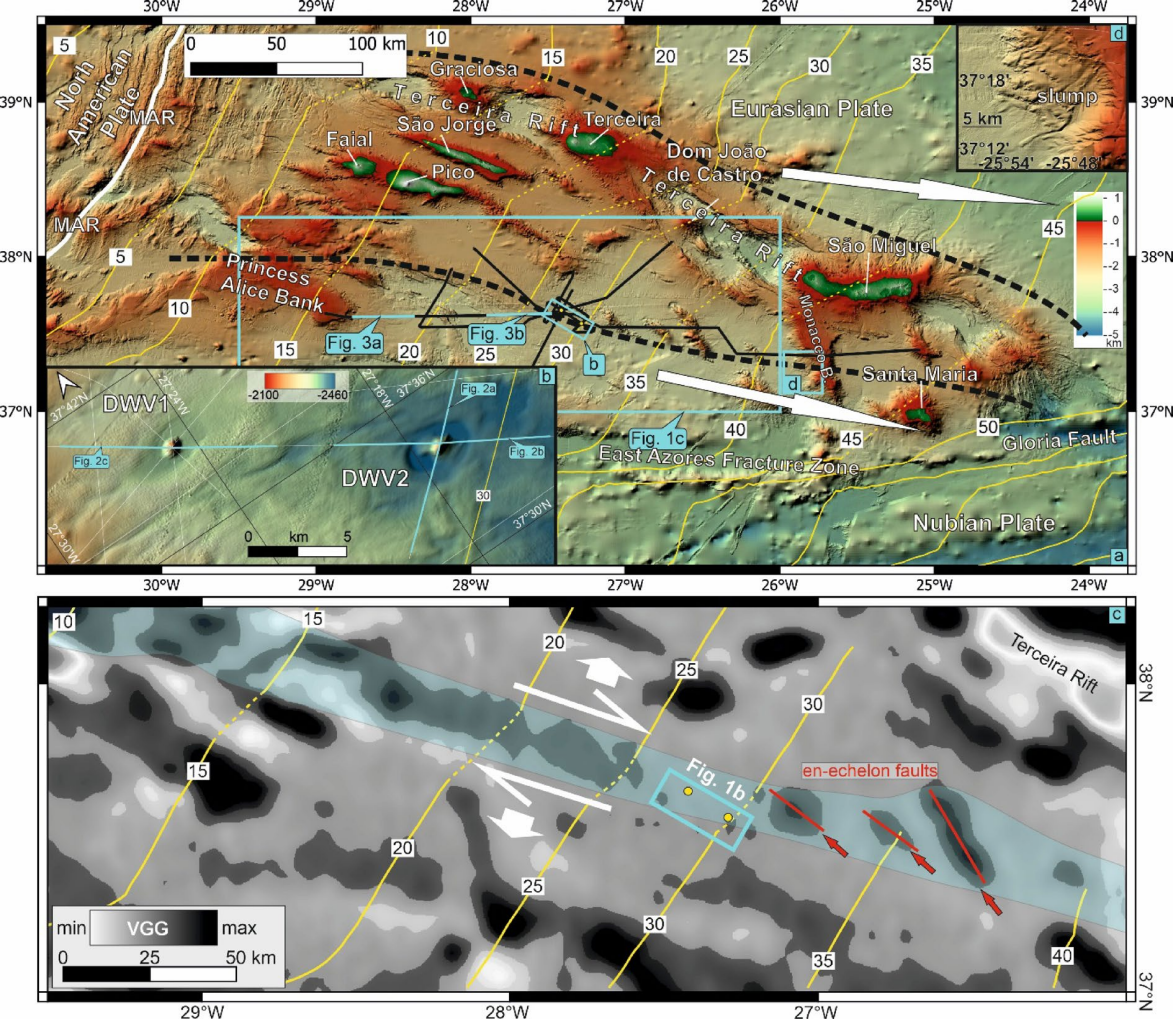
(**a**) Bathymetry of eastern Azores Plateau^49^. Black lines show seismic reflection profiles. Isochores of crustal ages in 5 Myrs intervalls in yellow^50^. Dashed black lines delimit the northern and southern extent of a diffuse plate boundary, and the white arrows indicate slightly diverging GPS-vectors of the Nubian and Eurasian Plate^29^ (length not to scale). The bluish area covers a positive vertical gravity gradient (VGG) anomaly along the southern boundary of the plate boundary. DWV: Deep water volcano; MAR: Mid-Atlantic Ridge. (**b**) Blow-up of DWV1. (**c**) Blow-up of DWV2. (**d**) Vertical gravity gradient (VGG)^51^. White arrows indicate transtension along diffuse plate boundary^29,32^. Yellow lines in yellow as in (**a**). Red arrows point towards volcanic ridges. See Supplementary Fig. S1 for enlarged version. (**e**) Blow-up of a slump on the SW flank of the Monacco Bank. Maps were created using QGIS v.3.40.

Spatially isolated DWVs occur across the central Azores Plateau^8,20^ Fig. 1), which formed from a mantle melting anomaly between 10 and 4 Ma^21–24^. This melting is attributed not only to thermal upwelling but also to a volatile-enriched mantle source^25,26^.

The East Azores Fracture Zone (EAFZ) defines the southern plateau margin and represents the fossil trace of the Gloria Fault on the Nubian Plate (Fig. 1)^27^. Initially connected to the Mid-Atlantic Ridge (MAR) at a ridge–fault–fault triple junction, the EAFZ migrated northward during the Oligocene–early Miocene, forming the Azores Microplate^28^. Today, the Nubian–Eurasian plate boundary is diffuse^29–31^ and extensional due to the anticlockwise rotation of the Nubian Plate^32^. Its northern limit follows the Terceira Rift^33^, while the southern margin aligns with a lineament of high vertical gravity gradient (VGG), volcanic ridges, the southern Monacco Bank, and Santa Maria Island (Fig. 1; Supplementary Fig. S1).

Submarine volcanism between 10–4 Ma and 2–0 Ma was widespread, with younger eruptions concentrated along structural weaknesses such as the Terceira Rift (< 1 Ma)^34^. By ~ 2 Ma, the rift was well developed, characterised by the accumulation of extrusive rocks^21,35^. During the last 1 Ma, recent volcanism occurred all along the diffuse plate boundary building up the eastern island of Sao Miguel^36^, and elongated volcanic ridges and islands in the Central Azores^34,37–39^.

Submarine volcanism and hydrothermal activity are reported throughout the plateau^6,8,9^. Morphometric analyses of cones down to 3200 m water depth show no seismically resolvable sediment cover, with many edifices being fault-controlled or associated with major volcanic structures such as the East Formigas High^8,40^. Cone widths reach up to 2400 m and heights up to 500 m above the seafloor, with no systematic correlation between geometry and water depth beyond 300–400 mbsl. Seismic profiles reveal chaotic internal reflections within the cones and concave-upward laminated reflections along their flanks, terminating as downlaps at the seafloor^8,34^. The largest volcanic edifice, Dom João de Castro Seamount, rises from > 2000 mbsl to just below sea level along the ultraslow-spreading Terceira Rift axis^40^. Geochemical data indicate ongoing, though waning, hydrothermal activity both within the rift and across the central plateau^41,42^.

In this study, we integrate multibeam and high-resolution seismic data from two DWVs in the central Azores Plateau (Fig. 1) to assess their eruption styles. The recently published results from IODP Expedition 398 allow, for the first time, the validation of interpretations of seismic reflection patterns regarding the extent of fragmentation of lava and eruptive products, even in the deep sea^43–48^. We show that the absence of a visible crater does not exclude an early explosive phase. Our study suggests that a significant part of the volcanism that shaped the lower part of the DWVs was explosive, likely driven by magmatic degassing. Volatile emissions during such submarine eruptions may thus be greater than initially thought, with possible significant environmental consequences.

## Results

### Bathymetry and vertical gravity gradient

The bathymetry (50 m cell size)^49^ highlights two DWVs along with surrounding depressions (Fig. 1b, c). The DWVs, named DWV1 and DWV2, are situated within the central Azores Plateau, outside of any topographically discernible grabens, but along the southern margin of the presently active diffuse plate boundary^29^. Here and in the following, we round the depth values to full 10 m due to the varying accuracy (0.2–0.5% of the water depth)^49^. The summit of DWV1 lies at 2220 mbsl, and its base is at 2450 mbsl (Fig. 1b). DWV1 has a basal diameter between 1230 and 1770 m, and a maximum slope angle of approximately 22–24°, while the NW and SE flanks are irregular. The diameter of the ring-shaped depression measures 3650 m (E-W) and 3240 m (N-S). The summit of DWV2 lies at 2170 mbsl, and its base extends to 2370 mbsl (Fig. 1c). Its diameter at the base is between 1290 and 1430 m. Like DWV1, its maximum slope angle is approximately 22–24°. The surrounding depression has a diameter of 4170 m (E-W) and 2650 m (N-S). Both depressions are slightly ellipsoid.

The age of the oceanic crust beneath DWV2 is ~ 28 Ma, while that beneath DWV1 is ~ 30 Ma^50^ (Fig. 1b, d). Both DWVs are located along the southern limit of the diffuse plate boundary (Fig. 1a). Along this zone, the vertical gravity gradient (VGG) reveals a SE-striking, continuous positive anomaly on crust aged 10–25 Ma^51^ (Fig. 1d). Extending SE and on crust aged 30–37 Ma, this anomaly aligns with volcanic ridges and related positive VGG anomalies. In the further extension and along the southern diffuse plate boundary, a slump is evident on the Monaco Bank’s southern flank (Fig. 1e), while the island of Santa Maria is situated further east along the anomaly (Fig. S1).

### Seismic stratigraphic units

**Fig. 2:**
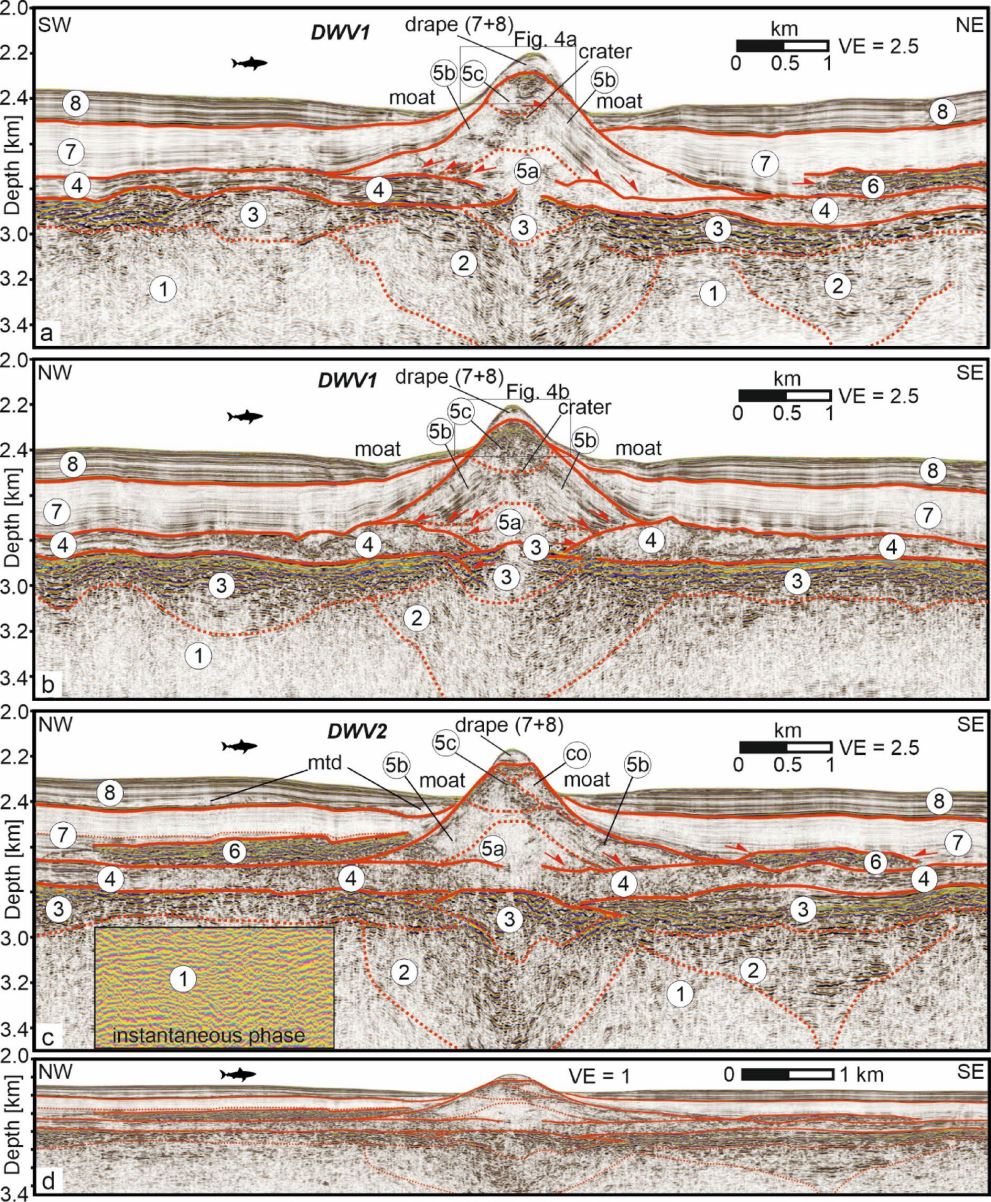
Interpreted seismic depth sections from deep water volcanoes (DWV). The profile in (**a**) and (**b**) are cross sections over the eastern DWV2 (crustal age ca. 30 Ma^50^). The crustal age of DWV1 is ca. 28 Ma^50^. For locations see Fig. 1b,c. 2c is reproduced in 2d but without vertical exaggeration. Numbers correspond to seismic units. For uninterpreted sections, see Supplementary Fig. S2. co: cone; mtd: mass transport deposit.

The internal architecture of DWV1 was analyzed using two intersecting seismic depth profiles crossing its summit (Fig. 2a, b) and compared with a profile across DWV2 (Fig. 2c). In order to better visualize the internal structures of the DWVs, the seismic sections in Fig. 2a–c are vertically exaggerated by a factor of 2.5, while the section from Fig. 2c is reproduced in Fig. 2d without vertical exaggeration to visualize the actual geometric conditions. Uninterpreted seismic sections are shown in Supplementary Fig. S2. For the description, we use the term “seismic unit” for a mappable interval of seismic reflections whose characteristics differ from those of adjacent seismic units, independent of their petrological or geochemical nature. We use a standard terminology for the description of the seismic units^52^, as summarized in Table 1.

**Table 1:** Description of identified seismic units by using seismo-stratigraphic standard terms^52^ and their interpretation. Each data segment is 1 km wide and 200 m high. The positions of each data segment are shown in Figure S2.

Unit	Image	Reflection characteristics	Interpretation
8		Strong amplitudes; parallel to slightly wavy reflections. Drapes underlying Unit 7. Occasionally with extensive chaotic facies	Hemipelagites with intercalated ash from subaerial volcanism^36–39^
7		Weak amplitudes; parallel to slightly wavy reflections. Locally disrupted every few hundred metres. Drapes underlying units; sharp contrast to overlying Unit 8	Hemipelagites
6		High amplitudes; stratiform reflections. Locally developed. Gradual transition to underlying Unit 4; sharp transition to overlying Unit 7	Lava flows^13,54,55,59–61^
5c		Upward-increasing amplitudes and reflection continuity; chaotic in the lower part. Overlies and fills a depression at the top of Unit 5b	Upwards coarsening volcaniclastics, possibly lava (this study)
5b		Variable amplitudes; upwardly concave and outward-converging continuous reflections, intercalated chaotic reflection packages. Downlapping underlying units; crater at the top	Volcaniclastics, formed by explosive eruptions^8,43–47,62–64,69–72,75–78^
5a		Weak but variable amplitudes; granular in the centre, chaotic or partly outward-converging; overall mound-shaped geometry. Gradual transition to underlying Unit 4 and overlying Unit 5b	Volcaniclastics^8,69–73^, possibly hydrothermal deposits^62,74^
4		Variable amplitudes, generally higher than those of Unit 7; sub-parallel to chaotic reflections beneath the DWVs, becoming continuous and parallel with increasing distance	Hemipelagites, overprinted by incipient volcanic eruption (this study)
3		High amplitudes; lower part stratiform, upper part mainly continuous. Gradual transition to overlying Unit 4	Lava flows^13,54,55,59–61^
2		Strong amplitude variability; stratiform; upwardly concave reflections, funnel-shaped unit, nested within Unit 1 where underneath DWVs and to the side	Hemipelagites overprinted by ascending magma and fluids (this study)
1		Weak but variable amplitudes; stratiform reflections. Lower boundary not imaged. Gradual transition to nested Unit 2 and overlying Unit 3	Hemipelagites

Unit 1 occurs approximately 500 m below the seafloor and is characterised by small but variable amplitudes and stratiform reflections. Its lower boundary is not imaged, and it shows a gradual transition to the nested Unit 2 above and to the overlying Unit 3 (Fig. 2a–c). The instantaneous phase confirms the reflection geometry independently of reflection amplitude (Fig. 2c).

Unit 2 forms a downward-converging, funnel-shaped structure nested within Unit 1, with a maximum width of 3–4 km at its upper boundary (Fig. 2a–d). The unit exhibits strong amplitude variability with stratiform, upwardly concave reflections. The lateral transition between Units 1 and 2 is gradual. Where Unit 2 is present beneath the DWVs, internal reflections dip inward, with their deepest points directly below the volcanic summits. The upper boundary forms a depression about 100 m deep and 1 km wide, while the base of Unit 2 is not imaged.

**Fig. 3:**
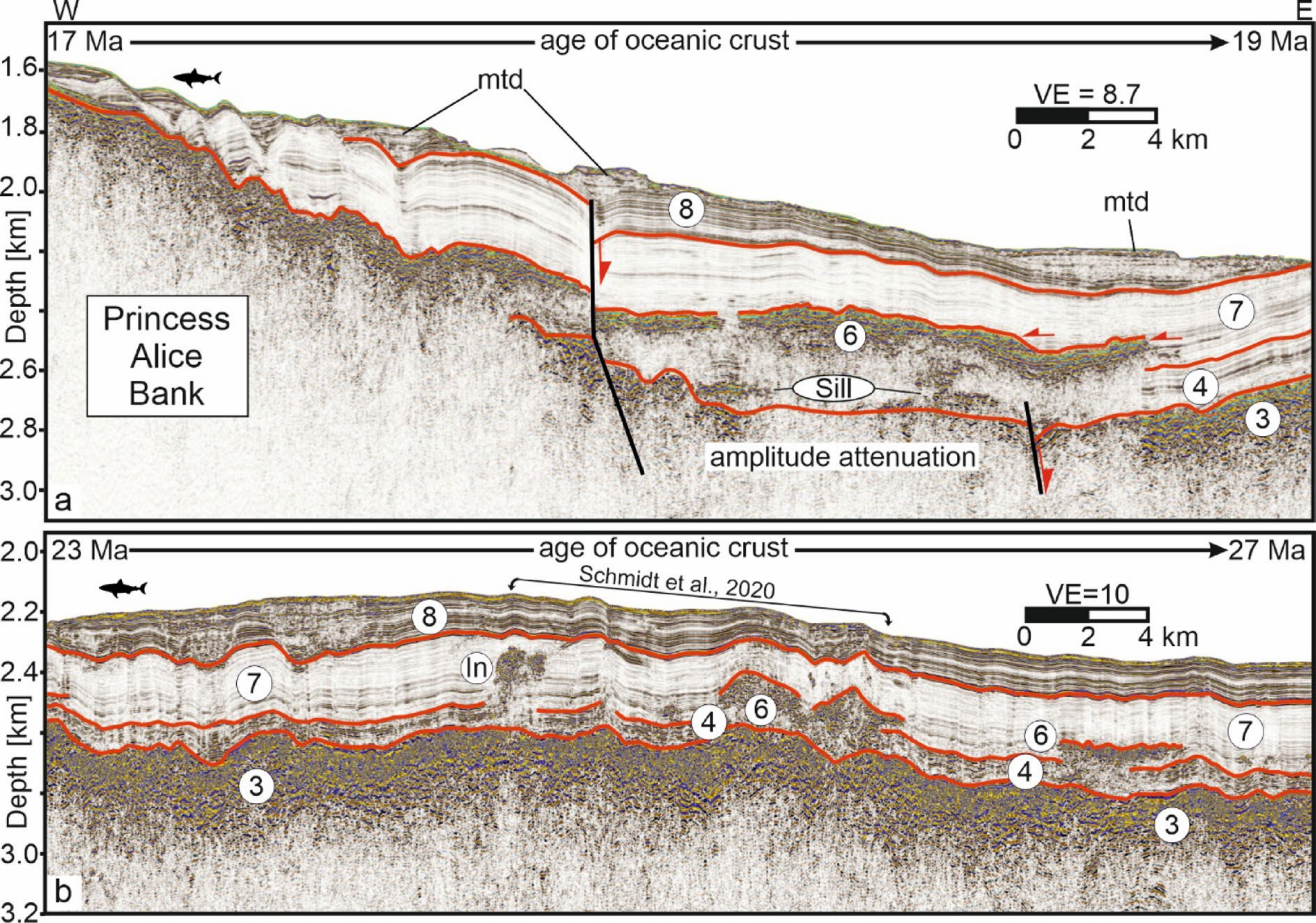
Interpreted seismic depth sections. For locations, see Fig. 1a. Numbers correspond to seismic units. I: Intrusion. For uninterpreted sections, see Supplementary Fig. S3. In: intrusion; mtd: mass transport deposit.

Unit 3 is 100–200 m thick and marked by high amplitudes, with a stratiform lower part and a mainly continuous upper part. It consistently occurs at current depths around 2800 mbsl and about 400 m below sea floor (Fig. 2a–c). Unit 3 can be traced westward to the Princess Alice Bank (Fig. 3), increasing in thickness from ~ 100 m east of the DWVs to ~ 200 m in the west. Beneath the DWV summits, Unit 3 is reflection-free and fills the depression in Unit 2.

Unit 4, approximately 100–150 m thick, overlies Unit 3 and displays variable amplitudes, generally higher than those observed in Unit 7 (Fig. 2a–c). Beneath the DWVs, reflections are sub-parallel to chaotic, becoming increasingly continuous and parallel with distance (Figs. 2, 3).

Unit 5a is characterised by low but variable amplitudes and an overall mound-shaped geometry. Reflections are granular in the central part and chaotic or partly outward-converging towards the flanks (Fig. 2a–c). The unit shows a gradual transition to the underlying Unit 4 and to the overlying Unit 5b.

**Fig. 4:**
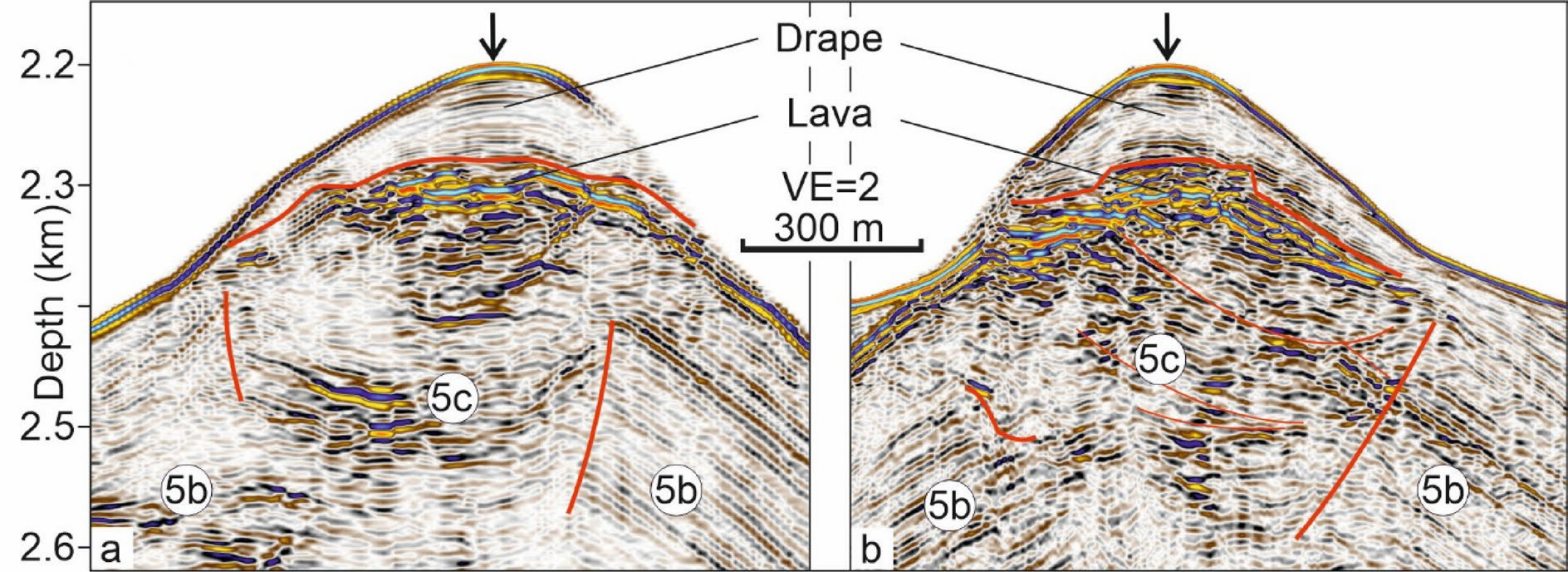
Interpreted seismic depth sections showing blow-ups from the top of DWV1. Numbers correspond to seismic units. Units 5b and 5c are separated by an upwards concave unconformity, interpreted as a crater. The upper boundary of 5c is upwards convex, interpreted as a lava cap. Internal unconformity within Unit 5c (**b**) are marked as thin red lines. Arrows mark the crossing points of (**a**) and (**b**). For uninterpreted sections, see Supplementary Fig. S4.

Unit 5b represents the main unit of the DWVs and is characterised by variable amplitudes and continuous, upwardly concave, outward-converging reflections with intercalated chaotic reflection packages. These reflections are nearly mirror-symmetric along the central axis in Fig. 2b. In the cross-section (Fig. 2a), the SW side shows granular to chaotic reflections with embedded high-amplitude horizontal patches. At DWV2, Unit 5b displays SE-oriented, upward-concave, outward-prograding reflections, contrasting with transparent or granular facies NW of the cone (Fig. 2c). The summit of Unit 5b is truncated in a crater-like manner at both DWVs (Figs. 2, 4). The upward-concave top of Unit 5b is best expressed at DWV1 (Figs. 2a, 4a).

The overlying Unit 5c is generally characterised by upward-increasing amplitudes and reflection continuity, with a chaotic lower part and an upward-convex upper boundary. Its internal reflection configuration varies with profile orientation: alternating weak and strong reflections are seen in Fig. 4a, while cross-sections (Fig. 4b) reveal internal unconformities. Within DWV2, a single mound labeled “co” occurs within Unit 5c (Fig. 2c). Units 5a–c together form a cone-shaped edifice approximately 4–4.5 km wide and 500–550 m high, yielding a height-to-width ratio of 0.11–0.14.

Unit 6 overlies Unit 4 northeast of DWV1 (Fig. 2a) and the outer flanks of DWV2 (Fig. 2c). It is ~ 100 m thick and marked by high amplitudes and stratiform reflections and occurs only locally. It shows a gradual transition to the underlying Unit 4 and a sharp transition to the overlying ~ 300 m thick Unit 7. Except for the upper DWV flanks, Unit 7 overlies Units 4, 5b, and 6. The parallel reflections drape the topography of the underlying units.

The ~ 100 m thick Unit 8 discordantly overlies Unit 7 and strong amplitudes with parallel to slightly wavy reflections and drapes the underlying Unit 7. Northwest of DWV2, Unit 8 contains weakly reflective, granular deposits labeled “mtd” (Fig. 2c). Unit 8 thins toward the DWVs, forming a circular moat depression (Figs. 1, 2).

Units 6–8 extend westward toward the Princess Alice Bank (Fig. 3), whereas Unit 6 is not continuous. The combined thickness of Units 4–7 remains broadly constant (400–500 m) but increases locally where Unit 6 is intercalated into Unit 7. Westward, Unit 6 loses its tabular geometry and develops a triangular form with high-amplitude reflections extending downward to Unit 3 (Fig. 3b). In the area described by Schmidt et al.^42^, amplitude anomalies within Unit 7 and deformation features in Unit 8 occur above Unit 6 (Fig. 3b). These reflection packages, labeled “In”, show characteristics similar to Unit 6. Additional anomalies below Unit 6 are labeled “Sill”. Westward, Unit 6 merges with the southeastern flank of the Princess Alice Bank, which exhibits comparable reflection patterns.

## Discussion

**Fig. 5:**
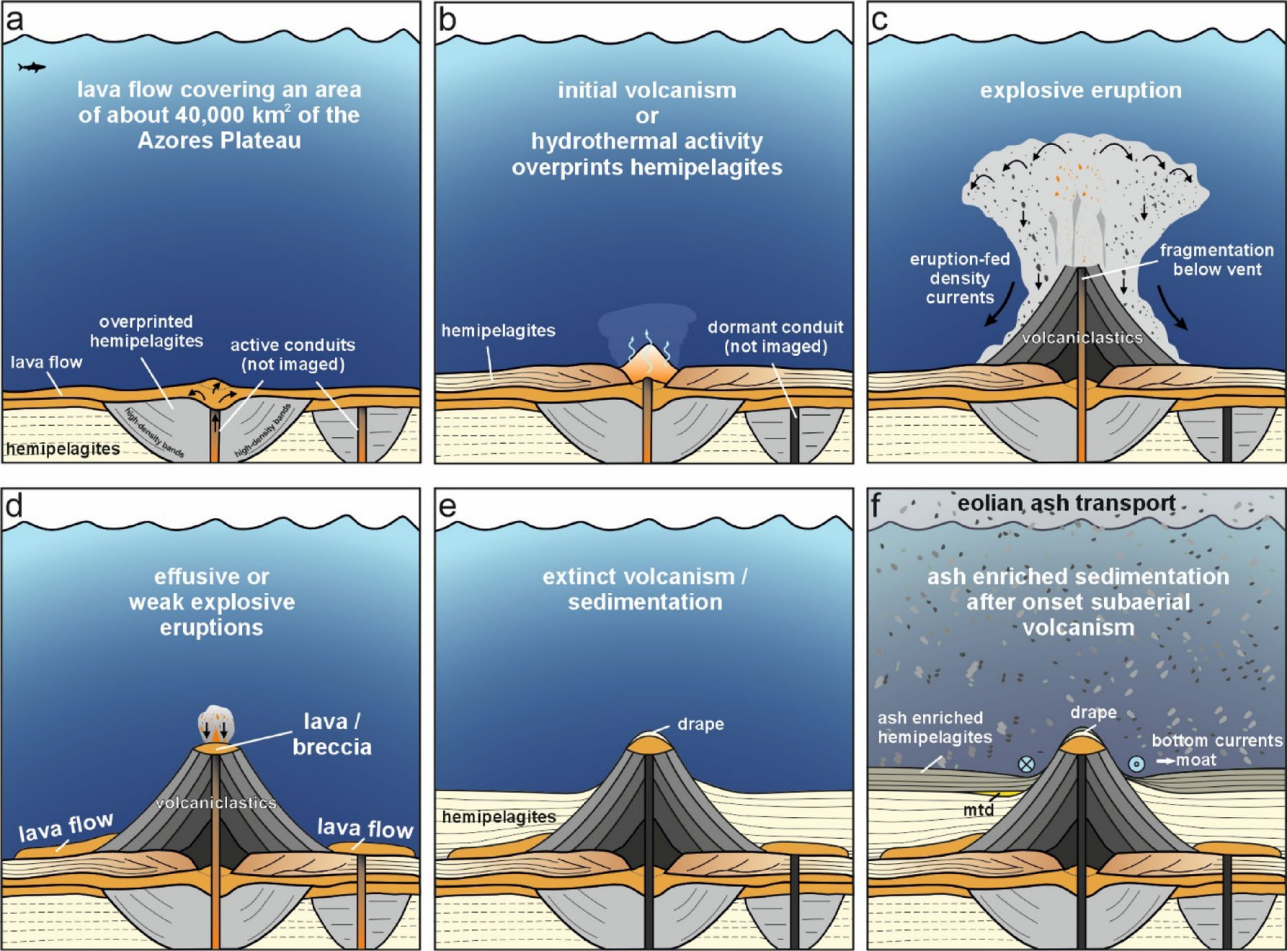
Conceptual sketch of the evolutionary stages of the submarine volcanoes (not to scale). See text for discussion.

Seismic data reveal a complex volcanic–sedimentary evolution of two DWVs on the central Azores Plateau. Since the origin of Unit 3 is needed first to justify the interpretation of Units 1 and 2, we start with the interpretation of Unit 3, which was previously interpreted as top oceanic basement^20^. However, although the crustal age increases from ~ 17 Ma at the eastern Princess Alice Bank to ~ 30 Ma near the DWVs, no systematic increase in the overburden thickness (Units 4, 7, 8) with crustal age is observable (Fig. 3), which also applies in the S–N direction between the EAFZ and the Terceira Rift (see Fig. 3 of Beier et al.^20^). It follows that Unit 3 was simultaneously deposited in its entirety (Fig. 5a). The high-amplitude reflection pattern also differs from typical oceanic crustal structures^53^. The hummocky reflection pattern of high reflection amplitudes like that of Unit 3 has been observed elsewhere and was interpreted as lava flows^13,54,55^. The flows laterally merge with the lower flank of the Princess Alice Bank, suggesting coeval emplacement during the already known transient mantle melting event at 10 Ma^20,25^.

Unit 3 extends from the Princess Alice Bank in the west at least to the DWVs (Fig. 3), as well as from the EAFZ in the south to the Terceira Rift in the north^20^. Due to the limited coverage of the Azores Plateau with reflection seismic profiles, the spatial extent of the lava flows cannot be accurately determined. In case the submarine Azores Plateau between 27°W to 29°W is fully covered by the tabular lava flows, this would correspond to an area of approximately 40,000 km^2^. Assuming an average thickness of 100 m, which is quite a conservative estimate, the volume of the tabular lava flows would be about 4000 km^3^.

To estimate the paleo–water depth at which the lava flows were emplaced, we considered the effects of sediment-load–induced isostatic adjustment, eustatic sea-level variations, and thermal cooling of the oceanic crust (see Methods). As we can only provide estimates, all water depth values are rounded to the nearest 100 m. Isostatic adjustment and sea-level variations result in a correction of approximately 100–300 m. Given that the lava flows (Unit 3) are currently located at ~ 2800 mbsl, this implies a minimum emplacement depth of ~ 2500 mbsl.

The crustal ages beneath the DWVs are 28–30 Ma (Fig. 1a). If Unit 3 formed at ~ 10 Ma, the crustal age at the time of emplacement was ~ 18–20 Ma. According to thermal subsidence models, this age difference should correspond to additional ~ 300 m of subsidence^56^. Accordingly, the present-day depth value of ~ 2800 msl would have to be reduced by ~ 600 m. Hence, the lava flows would have been emplaced at a minimum water depth of ~ 2200 m. However, no significant increase in the depth of Unit 3 (Figs. 2, 3). In addition, the overburden conformably overlies the lava flows which suggests that there is no significant residual thermal subsidence of the oceanic basement at the scale here considered. Age-dependent subsidence following the emplacement of the lava flows would have led to an upward-concave flexure, which in turn would have resulted in onlap termination of younger strata onto older sediments toward the MAR. The only termination of Unit 4 occurs where it onlaps Princess Alice Bank (Fig. 3a). Hence, it seems that thermal cooling–related subsidence is negligible. This may be explained, for example, by the assumed presence of a persisting shallow mantle anomaly, which is indicated by significantly reduced S-wave velocities at ~ 100 km depth beneath the eastern Azores Plateau^57^.

For an estimate of eustatic sea-level fluctuations and if we allow some uncertainty in the age estimate, e.g. 12–8 Ma, the eustatic sea level varied between − 19 and + 1 m^58^. This value falls within the stated uncertainties and is not taken into account further.

The estimated paleo-water depth of ~ 2200 m which considers thermal subsidence should therefore be regarded as a minimum estimate of the emplacement depth.

Units 1 and 2 lie between these lavas and above the basaltic basement with an age increasing from ~ 17 Ma in the west to ~ 30 Ma in the east. This configuration implies the presence of marine sediments, such as hemipelagites (Fig. 5a). Lava flow sequences such as Unit 3 are known to attenuate seismic energy significantly^59–61^, which explains the discontinuous reflection pattern with varying reflection amplitudes.

The funnel-shaped Unit 2 underlies both DWVs (Fig. 2), implying a link to magma transport feeding the volcanoes (Fig. 5a). Unit 2 also occurs beneath Unit 3 in areas without inward-dipping reflections, likely due to geometric effects of the seismic cross-section. Funnel-shaped features beneath Miocene volcanic edifices, somewhat similar to Unit 2, have been observed and drilled in the Bass Basin between southern Australia and Tasmania, as well as in the Maahunui Volcanic System in the Canterbury Basin offshore New Zealand^62–64^. In both cases, the seismically imaged funnel-shaped structures were interpreted as diatremes formed during explosive eruptions. If this interpretation were applicable to Unit 2, it would imply that Unit 2 records an initial explosive volcanic phase. However, diatremes are excavational structures formed by highly energetic processes and are typically associated with the deposition of tuff rings along their margins. Unit 2, in contrast, exhibits a gradual transition to Unit 1 and lacks any surrounding eruption products. We therefore consider a highly energetic, explosive process as the origin of Unit 2 to be unlikely. Instead, the inward-dipping high amplitude reflections may result from magmatic intrusions (sills) or high-density bands created by crystallization processes as observed in fossil shallow conduits^65^. Other explanation for the upward concave reflection curves may be (i) from subsidence above a shallow magma reservoir not imaged in the data, (ii) from an old transtensional strike-slip fault compatible with formation of an extensional syncline or a gravity collapse structure, or (iii) from sediment compaction caused, e.g., by dewatering in the vicinity of ascending magma and associated heat. More detailed investigations will require further research activities, i.e. a denser grid of seismic profiles.

Well-stratified units such as Unit 4 are interpreted as hemipelagic deposits consisting of fine-grained biogenic and terrigenous material accumulated through vertical settling and slow lateral advection^66^. Deformed or chaotic zones within Unit 4, beneath or adjacent to the DWVs, are interpreted as the consequence of the incipient eruption and emplacement of the overlying DWVs (Units 5a–c; Fig. 5b). Unit 4 represents about one-fifth of the sedimentary sequence above Unit 3. Assuming a constant sedimentation rate and that the overlying deposits were emplaced within 10 Myr, the ca. 100 m thick Unit 4 would have been deposited in roughly 2 Myr, implying a sedimentation rate of ca. 5 cm/kyr. The estimated 5 cm/yr are at the lower end of the range of 4–11 cm/kyr, which was determined for various locations at the Azores Plateau^67,68^.

Seismic Units 5a–c (Figs. 2, 4) represent the structural components of the cone-shaped DWVs, with Unit 5a marking the initial stage. The absence of coherent reflections within Unit 5a, which represents the lowermost mounded feature of the DWVs (Fig. 2), rules out the presence of coherent volcanic rocks such as lava flows. The weak reflection amplitudes indicate low contrasts in acoustic impedance, as is the case with highly fragmented volcaniclastic materials, generated by an early phase of an explosive eruption^8,69–73^. However, Unit 5a does not necessarily have to be of volcanic origin. The cross-section of Unit 5a (Fig. 2a, b) reveals a geometry, size, and reflection characteristics that were previously interpreted as hydrothermal vent complexes^62,74^. Based on the seismic images, it is not possible to distinguish between the two explanations. Regardless of whether Unit 5a is of volcanic or hydrothermal origin, its formation has locally overprinted Unit 4 to such an extent that Unit 4 is no longer seismically distinguishable from Unit 5a above the conduits (Fig. 5b).

Coherent, smooth, low- to medium-amplitude upwards concave reflections along the flanks of marine volcanic edifices such as Unit 5b have previously been interpreted as volcaniclastic deposits resulting from explosive eruptions^8,69–72,75^, which is why we also interpret this unit as volcaniclastic in nature. That a reflection character like that observed in Unit 5b actually represents volcaniclastic deposits has so far been verified through scientific drilling during IODP Expedition 398 or crater-wall sampling only in intermediate to felsic back-arc settings^43–48,62–64,76–78^. The stratification may have resulted from pulsating, potentially polygenetic eruptions and the subsequent deposition of fining-upward sequences^48,77^. Since the reflection characteristics of volcanic deposits (or any other geological structure) depend solely on contrasts in acoustic impedance and depositional architectures and not on the magma’s chemical composition, the sampling results and visual observations validate our interpretation of Unit 5b as highly fragmented volcaniclastic in origin. We interpret the rotationally symmetric and truncated upper boundary of Unit 5b as a volcanic crater formed by explosive eruptions, since there is no evidence of collapse beneath it. This interpretation is further supported by the morphological parameters of the DWVs. The height-to-width ratio of 0.11–0.14 observed here is consistent with shallow-water volcanoes formed by explosive eruptions along the Reykjanes Ridge south of Iceland, where ratios of 0.07–0.13 have been reported for edifices up to 2 km in width^77^. Broader shallow-water volcanoes typically display lower height-to-width ratios, which is attributed to lateral spreading of the eruptive plume at the sea surface^77^. The volcanoes investigated along the northern Reykjanes Ridge are relevant for the DWVs studied here, as the explosivity of the eruptions—which produced volcaniclastic deposits with similar seismic reflection characteristics—is documented by eyewitness observations. If the top of Unit 4, currently located at ~ 2600–2700 mbsl, is taken as the initial depth of the explosive eruptions, these eruptions must have initiated at a minimum depth of ~ 2000 mbsl. Given the negligible amount of thermal subsidence, the initiation depth was likely up to ~ 300 m greater at ~ 2300 mbsl (see Method chapter).

An intense lava fragmentation of the lava creating volcaniclastics of the here studied DWVs can be explained by different processes. Elevated magmatic CO₂ contents^25,26,79^ may have increased the critical pressure of magma–water–CO₂ mixtures^80,81^, enabling explosive degassing at > 2 km depth^10–13^. Alternatively, pressure-insensitive fuel–coolant interactions (IFCI) between magma and seawater^82^ could have driven the explosive eruptions. A third possibility is spatter quenching during low-energetic explosive eruptions^3^. However, those low energetic eruptions would presumably not lead to wide-spread stratified deposits, and the presence of craters supports the interpretation of high-energetic explosive eruptions, whether driven by rapid volatile expansion or IFCI. The deposition of the volcaniclastic material then occurs as it settles from the eruption cloud^77^ onto the flanks of the DWV (Fig. 5c).

The significance of elevated magmatic CO₂ contents for the critical pressure of magma–water–CO₂ mixtures, as indicated by phase diagrams^80,81^, provides a mechanism that has received little attention in the literature for explaining explosive eruptions and the resulting glassy lava fragments at water depths of several kilometres. Notably, evidence for explosive deep-water volcanism has so far been described primarily at mid-ocean ridges in the Arctic Ocean (Gakkel Ridge^10^) and along or near mid-ocean ridges in the Pacific^11–13^, i.e., in basaltic lavas. However, sediment samples have also revealed glassy lava fragments along the Blanco transform fault, and in the Fiji back-arc basin^11^.

The craters are filled with the chaotic Unit 5c (Figs. 2, 4, 5d). Reflection packages of higher amplitudes within that unit exhibit characteristics comparable to those of the lava flows and are interpreted accordingly. The weaker reflecting packages may represent volcaniclastic material or hemipelagites. In either case, the eruption plume did not rise high within the water column, so that the eruptive products did not overspill the crater rims with seismically resolvable thickness but instead accumulated within the craters. This observation indicates a significant decrease in eruptive explosivity, suggesting that toward the end of volcanic activity, lava extrusion became predominantly effusive. The decrease in explosivity can be attributed to the progressive depletion of the CO_2_ budget, which acts as a critical factor for magma ascent dynamics^83^. Due to its significantly lower solubility compared to H_2_O, CO_2_ is preferentially degassed during the early stages of an eruption, often following a transient fluxing event from deeper reservoirs^84^. As this CO_2_ supply is exhausted, the resulting change in the H_2_O–CO_2_ mixture shifts the fragmentation level within the conduit and reduces the overall gas volume fraction^85,86^. Alternatively, the magmatic system may have changed its composition from a more CO_2_ enriched silicic composition to a more mafic composition reducing the CO_2_ supply due to a lower degree of enrichment. Independent of the origin of the CO_2_ decrease, this decrease in the volatile-driven driving force lowers the mass discharge rate and may prevent the magma from reaching the critical strain rates required for brittle fragmentation^85^. Consequently, the exhaustion of CO_2_ facilitates a transition from explosive activity to effusive phases or eventual cessation, as the remaining magma lacks the internal pressure to sustain high-velocity ascent and fragmentation^87^. The infilling and burial of craters by lava flows or coarser, less fragmented lava if compared to the volcaniclastic deposits of Unit 5b may explain, why craters are not observed in bathymetric surveys of volcanic structures on the Azores Plateau^7,8^.

Due to its reflection characteristics, which closely resemble Units 3, we interpret Unit 6 as a package of lava flows that overlies the volcaniclastic flanks of DWV2 (Unit 5b; Fig. 2c; 5d). Geochemical analyses of sediments above Unit 6 west of the DWVs indicate ongoing hydrothermal activity linked to magmatic processes^41,42^, and these authors constrain Unit 6 to ~ 5 Ma, broadly coeval with the onset of island formation at Santa Maria^88–91^. The unit labeled “In” in Fig. 3b is also interpreted as volcanic (lava), but it is non-tabular and structurally more complex. Stratigraphically above Unit 6, this package records later volcanic activity on the eastern Azores Plateau, including intrusion into Unit 7.

After volcanic activity at the DWVs ceased, the hemipelagic sediments of Unit 7 accumulated, overlain by Unit 8, which shows higher reflection amplitudes compared to Unit 7 likely caused by ash layers deposited after the onset of subaerial volcanism on the Azores Plateau starting ca. 1,3 Mya ago^36–39^ (Fig. 5e, f). Mass-transport deposits (MTDs; Figs. 2c, 3, 5e) suggest that the onset of subaerial volcanism was accompanied by mass wasting, probably triggered by earthquakes that remobilized the sediment drape. The circular depressions surrounding the edifices represent moat channels, similar to those south of São Miguel, interpreted as contour-current features formed by the return flow of the North Atlantic subtropical gyre^92^.

The sediment draped upper DWV sections protruding from the seafloor are ~ 1.5 km wide and ~ 0.25 km high (height/width ratio 0.17), consistent with bathymetric analyses of Azores Plateau cones (0.10–0.26)^8^ and cones near Pico Island^6^. Including the buried lower flanks, the volcanic core (Units 5a–c) reaches 4.0–4.5 km in width and 0.42–0.47 km in height, reducing the height/width ratio to 0.09–0.12. These results highlight that seismic imaging is essential for accurately determining volcanic geometry, and further comparative studies are needed to assess whether geometric parameters can reliably indicate volcaniclastics.

The two DWVs and the three volcanic ridges marked in Fig. 1 are located along the southern boundary of the transtensional diffuse plate boundary^29,32^. While the Terceira Rift and the NAFZ are characterised by low VGG values due to crustal extension compared to adjacent areas of the Azores Plateau, the transtensional plate boundary exhibits a positive VGG anomaly. We propose that extension of the 10–20 Ma oceanic crust facilitated sustained magma ascent, with intrusions or magmatic underplating generating the elongated positive VGG anomaly and promoting magma rise beneath the volcanic centers. The three magmatic ridges to the southeast, striking 307° to 327°, correspond to right-lateral transtensional tension gashes. These tectonic processes may also have triggered the collapse of the Monacco Bank^93^ and possibly the ~ 5.1 Ma collapse of Santa Maria^29,88,91^.

## Conclusions

High-resolution seismic reflection data provide new insights into the internal architecture of deep-water volcanoes on the Azores Plateau, allowing distinction between explosive and effusive eruption phases and their transitions. This is possible due to recent calibrations of seismic interpretations with IODP drill cores and ROV sampling in other volcanic regions, which have demonstrated that seismic reflection characteristics can reliably distinguish between volcaniclastic deposits from explosive eruptions and lava flows from effusive activity.

On the Azores Plateau, the data—together with previously published results^20^—indicate that around 10 Ma, lava flows up to 200 m thick covered an area of more than 40,000 km^2^ at ~ 2400 m water depth (Fig. 5a). After a non-volcanic interval, possibly involving hydrothermal vent activity, an explosive–effusive phase built volcanic edifices about 400 m high with craters ~ 500 m wide (Fig. 5b, c). Whether the explosivity was driven by expanding CO₂ or by induced fuel–coolant interactions remain uncertain. Given the pressure-dependent behavior of water–CO₂–magma mixtures, CO₂-driven mechanisms cannot be excluded. Subsequent less explosive or even effusive eruption products filled these craters (Fig. 5d). The post-volcanic sedimentation reflects both the onset of subaerial volcanism and bottom currents (Fig. 5e, f).

This study underscores the value of seismic reflection profiling for identifying explosive submarine volcanism, both through the imaging of infilled craters and the detection of reflection patterns typical of volcaniclastic deposits. Neither of these indicators can be reliably detected through conventional sampling of shallow deposits or bathymetric surveys alone. Consequently, explosive deep-water volcanic eruptions may be more common than previously assumed, both on the Azores Plateau and globally. As explosive eruptions usually release a great amount of volatiles (including CO_2_)^2^, the systematic search for similar, yet under-explored, volcanic eruptions on ocean seafloors worldwide may provide important constraints for magma outgassing budgets and their potential climatic and environmental impacts.

## Methods

The data were collected during Expedition M113/1 aboard RV METEOR^94,95^ with a marine reflection seismic equipment^96^. The seismic source consisted of three GI- and one Mini-GI air pulser with a combined primary volume of 150 cubic inches, towed at a depth of 2.5 m behind the vessel. The data were recorded using a 144-channel digital streamer, towed at the same depth. Seismic data processing involved several steps, including frequency filtering (20–400 Hz), velocity analysis (every 50 CMPs), correction for spherical divergence and energy loss, normal moveout (NMO) correction, coherency filtering, stacking, migration, white noise removal, dip filtering, and fx-deconvolution. The depth conversion of the seafloor was performed using a seismic velocity of 1.5 km/s, which is accurate to within a few percent. For the depth conversion of the sedimentary cover, we applied an interval velocity increasing from 1.6 to 1.9 km/s from the seafloor to the lava flows, with an average velocity of 1.8 km/s. In order to estimate possible errors, we assumed a minimum and maximum average velocity of 1.6 and 2.0 km/s, so the velocity has an accuracy of ± 11%. The TWT interval of the sediment package adjacent to the volcanic cones comprises approximately 0.5–0.6 km^20^, corresponding to 0.4–0.6 km thickness. A velocity of 3 km/s had been used for the depth conversion of the lava flows. Bathymetry and VGG data are publicly available.

For an estimate of the paleo-water depth of the volcanic eruptions we estimate the paleo-depth of Unit 3 by considering sediment load, crustal cooling and eustasy. A straightforward consideration of subsidence caused by sediment loading with a thickness d_s_ and a specific density ρ_s_ involves assuming an equilibrium depth beneath the Earth’s crust following subsidence^97^. Before subsidence, the Earth’s crust is overlain by water with a thickness d_w_ and a rounded specific density ρ_w_ = 1 Mg/m^3^ and underlain by mantle material with a thickness S and a specific density ρ_m_ = 3.3 Mg/m^3^. The formula for subsidence S, neglecting the decompaction of the underlying layers and calculating in 1D, is as follows^97^:

$$S = d_{s} \frac{{\rho_{s} - \rho_{w} }}{{\rho_{m} - \rho_{w} }}.$$

Based on core and logging data from the Christiana-Santorini-Kolumbo Volcanic Field in the Aegean (Mediterranean) Sea (IODP Exp. 398)^75^, we estimate the mean bulk density of the 0.4–0.6 km thick sediment layer between 1.65 Mg/m^3^ (pure ash) to 1.9 Mg/m^3^ (pure marine ooze). Hence, the subsidence amounts to 30–40% of the sediment thickness. Consequently, back stripping of 0.5–0.6 km of sediment results in an uplift of 0.15 to 0.24 km.

Generally, oceanic crust would be expected to subside as it cools over time^56^. The crustal age of the DWVs is 28–30 Ma. If Unit 3 developed 10 Ma, the crustal ages were 18–20 Ma when Unit 3 was emplaced. Unless the thermal cooling was delayed by magmatic processes, it led to a subsidence of approximately 0.3 km^56^.

## Supplementary Information

Below is the link to the electronic supplementary material.

Supplementary Material 1

## Data Availability

The seismic data shown in Figs. 2–4 can be downloaded from: Friedrich, A., & Hübscher, C., 2025. Multi-channel seismic depth sections from RV Meteor expedition M113 [Data set]. 10.5281/zenodo.15696584. The bathymetric data can be downloaded from: Hübscher, C., Beier, C. 2022a. Multibeam bathymetry processed data (EM 120 echosounder & Kongsberg EM 122 dataset compilation) of RV METEOR during cruise M79/2, M113/1 & M128, Azores Plateau between the Terceira Rift and the East Azores Fracture Zone, doi: 10.1594/PANGAEA.945528. North Atlantic Ocean, PANGAEA. EMODNet data are available from European Marine Observation Data Network (EMODnet) Bathymetry. https://emodnet.ec.europa.eu/geoviewer . VGG data are available from Sandwell, D.T., Harper, H., Tozer, B. & Smith, W.H., 2019. Gravity field recovery from geodetic altimeter missions. Advances in Space Research 68(2), 10.1016/j.asr.2019.09.011. Downloaded 2023-08-25 from https://www.generic-mapping-tools.org/remote-datasets/earth-vgg.html.
