# Supplementary material for: Seismic imagery from volcanoes on the Azores Plateau implies that explosive deep-water eruptions are more common than previously thought
Source: Sci Rep. 2026 May 13;16:15066. doi: 10.1038/s41598-026-53050-0 (PMC13172439; doi:10.1038/s41598-026-53050-0)
Supplement: Supplementary file 1 — Supplementary Material 1 [file 41598_2026_53050_MOESM1_ESM.docx]

**Seismic imagery from volcanoes on the Azores Plateau implies that explosive deep-water eruptions are more common than previously thought**

*Christian Hübscher^1*^, Annalena Friedrich^1^, Jonas Preine^2,3^, Christoph Beier^4^, Anthony Hildenbrand^5^, Paraskevi Nomikou^6^, Pedro Terrinha^7^, Benedikt Weiß^1,8^*

1) Department of Earth System Sciences, University of Hamburg, Hamburg, Germany.

2) National Oceanography Centre (NOC), Southampton, UK.

3) Department of Geology and Geophysics, Woods Hole Oceanographic Institution, Woods Hole MA, USA

4) Department of Geosciences and Geography, Research Programme of Geology and Geophysics (GeoHel), University of Helsinki, Helsinki, Finland.

5) GEOPS, Université Paris-Saclay, CNRS, Orsay, France.

6) Department of Geology and Geoenvironment, National and Kapodistrian University of Athens, Athens, Greece.

7) Department of Marine Geology and Georesources, Portuguese Institute for the Sea and Atmosphere (IPMA), Lisbon, Portugal. Instituto Dom Luiz (IDL), Lisbon, Portugal.

8) Current address: Bundesamt für Seeschifffahrt und Hydrographie (BSH), Hamburg, Germany.

* Corresponding author: christian.huebscher@uni-hamburg.de

**Supplementary Figures**


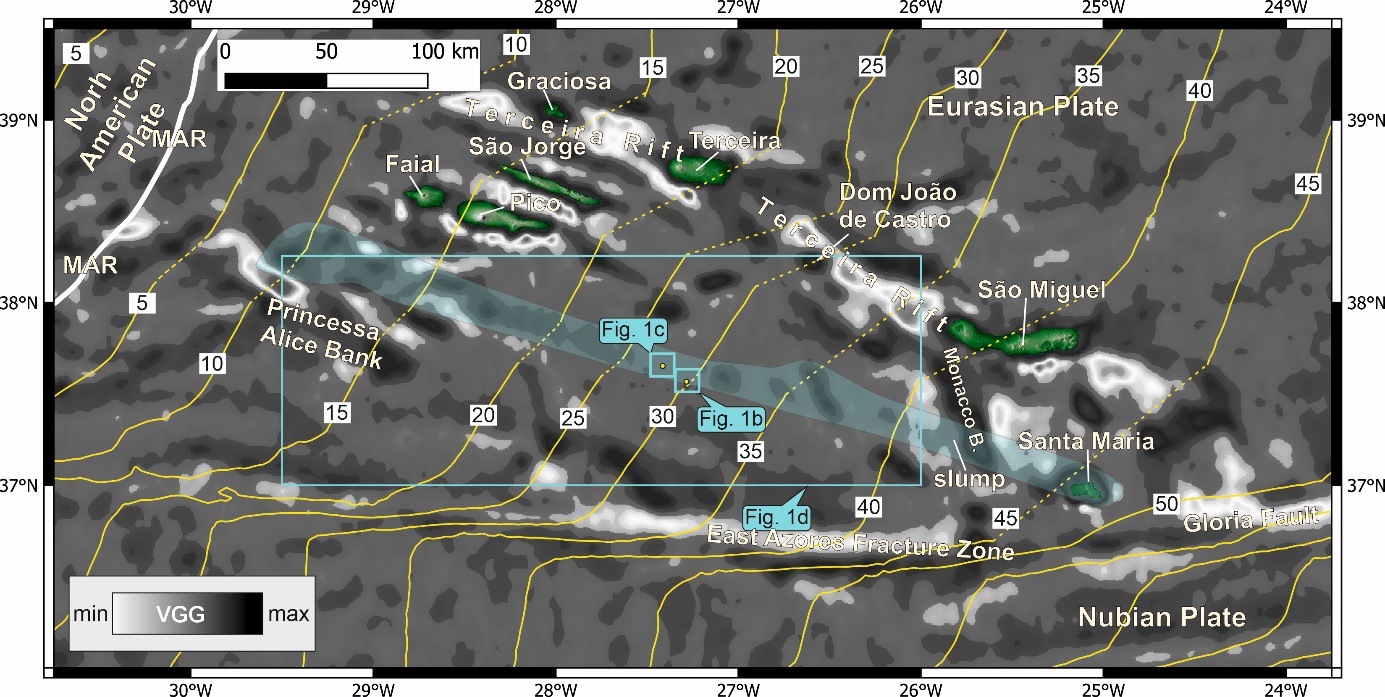


Supplementary Figure S1: Map of the vertical gravity gradient (VGG) of the central Azores plateau^51^. Yellow lines show isochores of crustal ages in 5 Myrs intervals (black numbers)^50^. The interpreted transitions across fracture zones are marked by dashed yellow lines. The bluish area covers an elongated and positive VGG anomaly along the dextral transtensional fracture zone along the southern boundary of the diffuse plate boundary^29,32^. MAR: Mid-Atlantic Ridge. Maps were created using QGIS v.3.40.


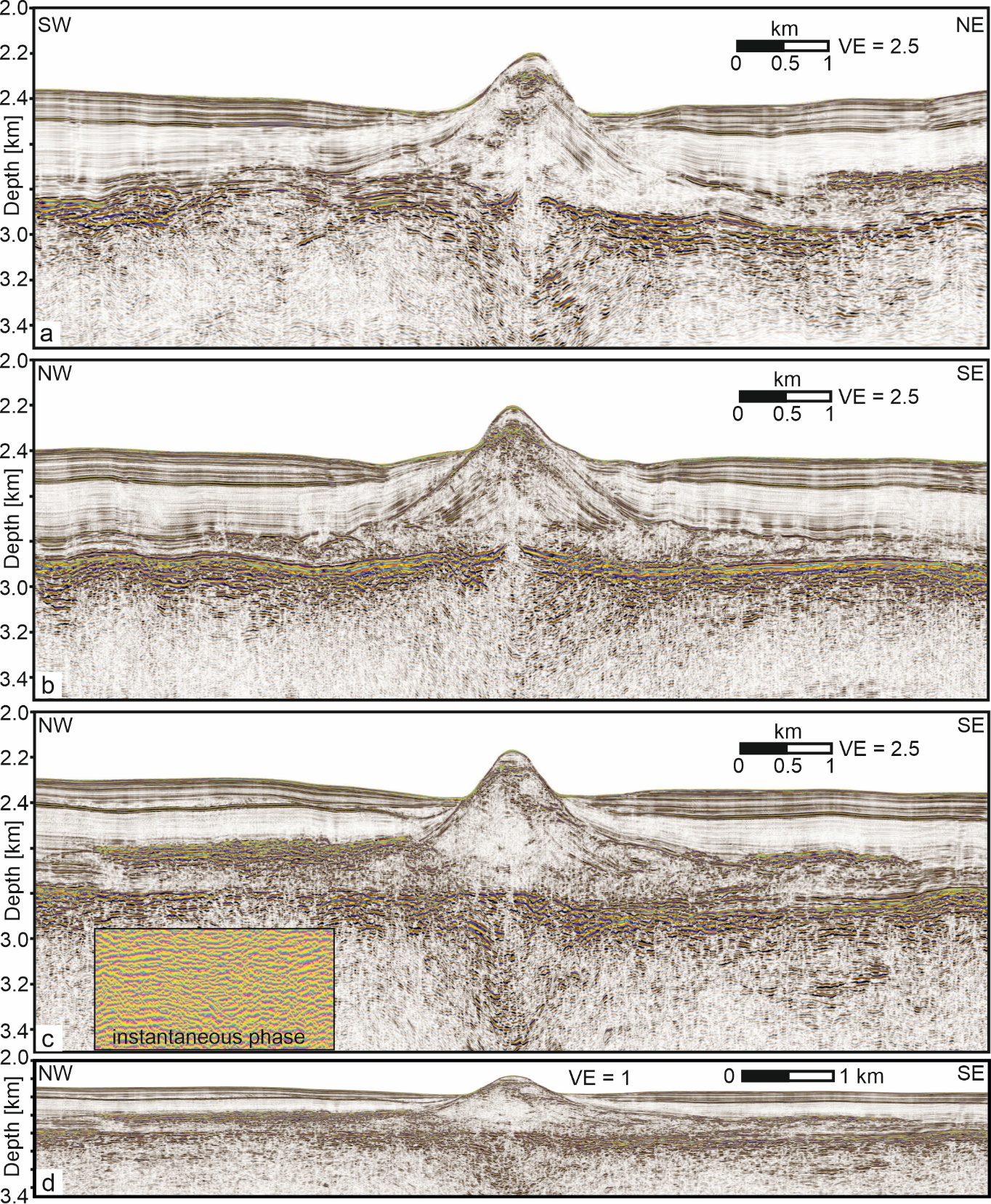
Figure S2: Seismic sections from Fig. 2 without interpretation.
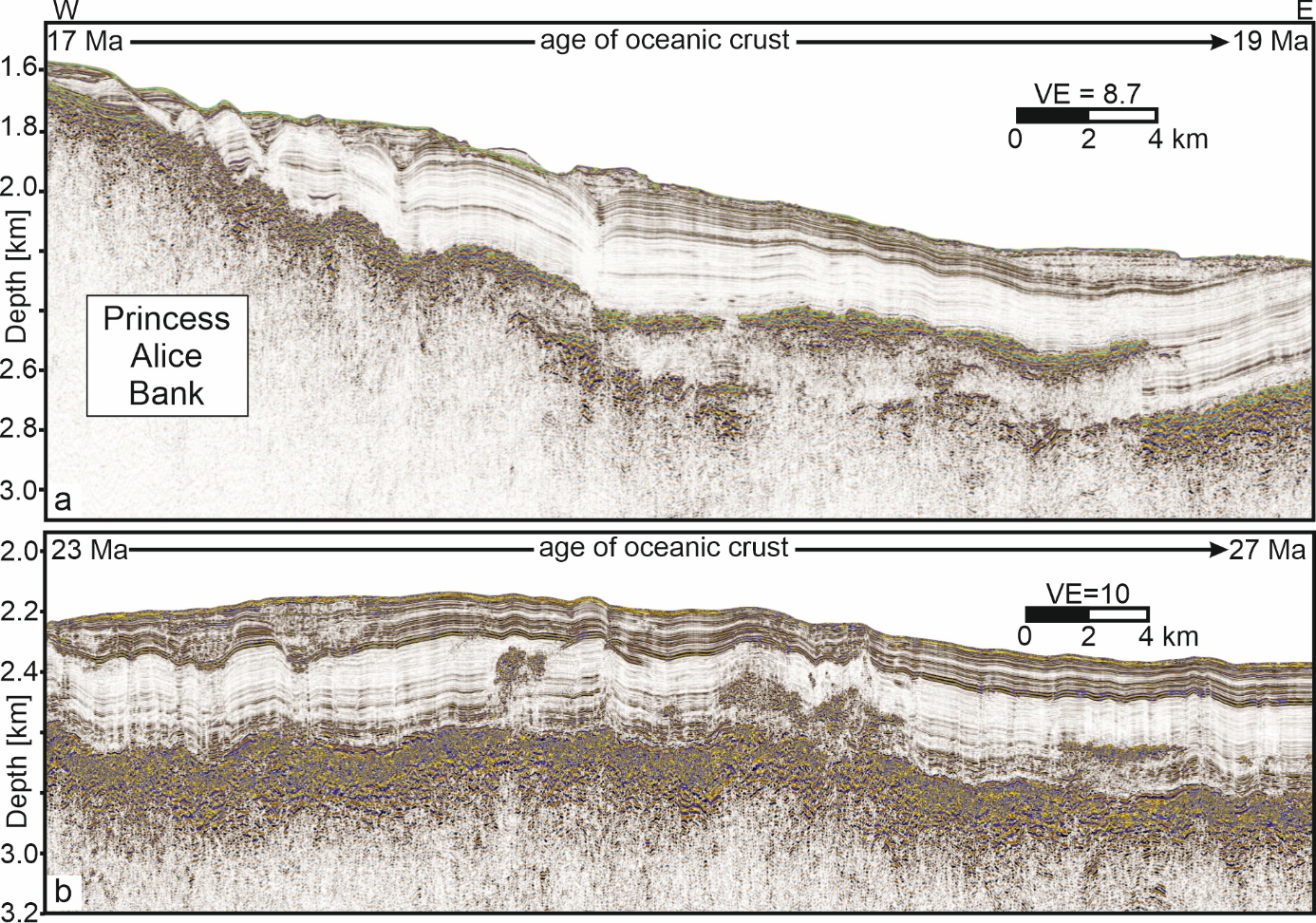


Figure S3: Seismic sections from Fig. 3 without interpretation.


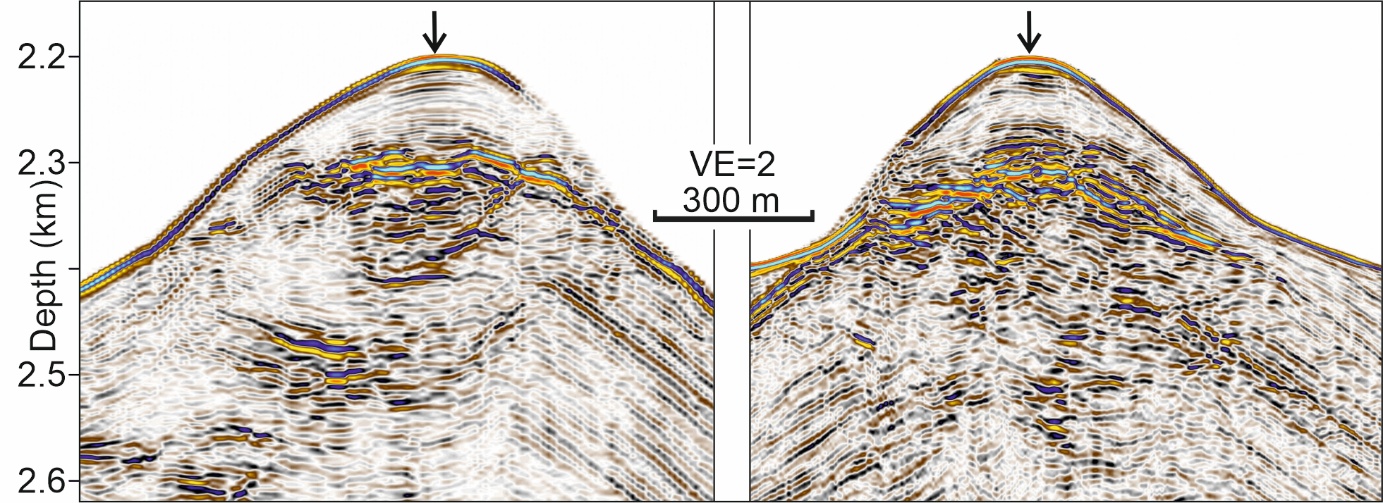


Figure S4: Seismic sections from Fig. 4 showing crossing seismic profiles over upper DWV1 cone without interpretation. Arrows mark the crossing points of a) and b).
